# Therapeutic patterns and migraine disease burden in switchers of CGRP-targeted monoclonal antibodies – insights from the German NeuroTransData registry

**DOI:** 10.1186/s10194-024-01790-7

**Published:** 2024-06-03

**Authors:** Ja Bin Hong, Heike Israel-Willner, Andreas Peikert, Peter Schanbacher, Viola Tozzi, Monika Köchling, Uwe Reuter, Bianca Raffaelli, NTD Study Group

**Affiliations:** 1 Department of Neurology, Charité Universitätsmedizin Berlin, Berlin, Germany; 2 Neurological Specialist Center Berlin (NFZB), Berlin, Germany; 3 Neurologicum Bremen, Bremen, Germany; 4 NeuroTransData, Neuburg, Germany; 5 Rewoso AG, Zurich, Switzerland; 6 Hochschule Furtwangen (HFU), Furtwangen, Germany; 7 Universitätsmedizin Greifswald, Greifswald, Germany; 8 Clinician Scientist Program, Berlin Institute of Health at Charité (BIH), Berlin, Germany; 9 NeuroCentrum, Grevenbroich & Dormagen, Germany

**Keywords:** Migraine, Prophylaxis, Calcitonin-gene-related peptide, Monoclonal antibody, Real-world experience

## Abstract

**Background:**

Monoclonal antibodies (mAbs) targeting the calcitonin gene-related peptide (CGRP) pathway have shown good efficacy in migraine prophylaxis. However, a subset of patients does not respond to the first mAb treatment and switches among the available mAbs. The goal of this study is to characterize the switching pattern of migraine patients treated with anti-CGRP(-receptor, -R) mAbs, and to describe the headache burden of those who did not switch, switched once, and switched twice.

**Methods:**

This study used real world data from the NeuroTransData Cohort, a registry of migraine patients treated at outpatient neurology clinics across Germany. Patients who had received at least one anti-CGRP(-R) mAb were included. Headache diaries were collected at baseline and during treatment, along with quality of life measures every three months. Results were summarized for the subgroups of patients who did not switch and those with one and two switches.

**Results:**

Of the 655 eligible patients, 479 did not switch, 135 switched once, 35 twice, and 6 three or more times. The ≥ 50% response rates for monthly migraine days were 64.7%, 50.7%, and 25.0% for the no switch, one switch, and two switches groups in their last treatment cycles, respectively. Quality of life measures improved for the no switch and one switch groups, but not for the two switches group.

**Conclusion:**

Patients who switched among anti-CGRP(-R) mAbs during the course of their treatment still benefited overall but to a lesser extent than those who did not switch. Treatment response in patients who switched twice was markedly lower compared to the no switch and one switch subgroup.

**Supplementary Information:**

The online version contains supplementary material available at 10.1186/s10194-024-01790-7.

## Introduction

Migraine is a debilitating neurological disorder with a high prevalence, particularly among otherwise healthy and working-age adults [1]. With the advent of calcitonin gene-related peptide (CGRP) targeted therapies, clinicians have new therapeutic options with a better efficacy and tolerability profile compared to standard oral preventive treatment [2, 3]. In Germany, erenumab entered the market in November 2018, followed by galcanezumab in April 2019, fremanezumab in May 2019, and eptinezumab in September 2022. While the majority of patients in clinical trials and real-world observational studies experienced an improvement, a subset of patients treated with anti-CGRP(-receptor, -R) monoclonal antibodies (mAbs) do not have a clinically meaningful response [4, 5]. Most migraine patients that receive anti-CGRP(-R) mAb in a real-world setting had no success with or have contraindications to multiple other classes of traditional preventive medications. Therefore, switching to another anti-CGRP(-R) mAb emerges as a frequently chosen therapeutic next step in non-responders to anti-CGRP treatment.

As the use of anti-CGRP(-R) mAbs becomes increasingly common in clinical practice, experience with switching among the three available subcutaneous antibodies, i.e. the anti-CGRP-R antibody erenumab and the anti-CGRP antibodies galcanezumab and fremanezumab, is growing. A treatment attempt with a second anti-CGRP(-R) mAb can eventually result in a clinically significant improvement in up to 45% of patients [6–8]. If no response was seen after the first switch, a second switch may still lead to a positive treatment outcome [9].

Patients who end up switching between different anti-CGRP(-R) mAbs are some of the most severely affected and challenging to treat migraine patients [7]. Studying the population of these difficult to treat migraine patients could help us understand and address the challenges faced by those patients, and to discover features that are more commonly associated with treatment resistance.

In this study, we sought to characterize the switching patterns and describe the headache burden of migraine patients who did not switch, switched once, and switched twice during treatment with anti-CGRP(-R) mAbs in outpatient clinics in Germany.

## Methods

### Study design

This is a real-world retrospective registry study based on the NeuroTransData (NTD) network. NeuroTransData (NTD) is a Germany-wide network of neurologists and psychiatrists founded in 2008. Currently, the NTD network includes 133 specialists in 66 practices serving about 600,000 outpatients per year on the indications of bipolar disorders, dementia, epilepsy, migraine (27,000 patients per year), multiple sclerosis (MS), Parkinson’s disease and schizophrenia. The NTD *Migraine registry* is a disease specific database digitally capturing demographic, medical history, and clinical variables from migraine patients in a real-world setting. It was started on January 01, 2017. Currently, it includes 7583 patients with migraine treated in 54 practices at the level of secondary care headache centers.

Of those, patients were included in our study if they had a diagnosis of migraine according to the International Classification of Headache Disorders (ICHD-3) diagnostic criteria [10], had at least 4 monthly migraine days (MMD) in the 4 weeks before initiation of treatment with anti-CGRP(-R) mAbs (baseline period), and if they received at least one of the anti-CGRP(-R) mAbs as prophylactic treatment for at least 28 days. For the remainder of this paper, we will define a month to be 28 days.

The index date was defined as the date of the first dose of the anti-CGRP(-R) mAb, and the baseline period as the 4-week interval prior to the index date. The follow-up period was defined as the time period from the index date to the end of treatment with the anti-CGRP(-R) mAb. We will refer to this period as one treatment cycle. The NTD treating physicians choose a start and end date of a therapy. If no end date was provided, the treatment cycle was censored at the extraction date. If there was a treatment gap of less than 3 months between treatment cycles with the same anti-CGRP(-R) mAb, the two cycles were merged, and the gap excluded from analysis. Treatment cycles with less than four weeks duration were excluded. If a patient received more than one type of anti-CGRP(-R) mAb, treatment cycles with each anti-CGRP(-R) mAb were considered separately. The baseline period was defined as the 28 days before the index date of the first therapy cycle.

Socio-demographic and clinical characteristics including age, employment status, education, body-mass index (BMI), comorbidities, type of migraine (episodic or chronic), acute and prophylactic medications used, the presence of medication overuse, and number of prior preventive treatment failures were collected at baseline. Medication overuse was defined as either more than 14 days per month for non-triptan medications, or more than 9 days per month for triptan and combination drugs. MMD, monthly headache days (MHD), and monthly days of acute medication (MDAM) were recorded at baseline and at each four-week interval after treatment initiation. A migraine day was defined as a headache day where the patient self-classified the headache as migraine, or if the pain level was ≥ 4/10, or a triptan was used as acute therapy, or the headache was one-sided, pulsating or worsened with physical activity, or an aura was present.

We additionally collected quality of life measures using the Migraine Disability Assessment Test (MIDAS) and the Headache Impact Test (HIT-6).

### Data collection

Demographic data, clinical history, patient-related outcomes and clinical information were captured in real time during clinical visits. The data comprised both optional and essential modules and fields. The essential modules and fields were clearly marked but were not compulsory. Data were entered digitally into the web-based registry either manually or directly from digital sources. All personnel underwent regular training to ensure the quality of data in the database. Patient-related outcomes were captured digitally by tablets or via a smartphone app and were automatically transferred into the database. For the purpose of this study, we retrospectively analyzed data that had been collected and stored in the NTD registry between January 1, 2017 and December 31, 2022.

### Statistical analysis

Continuous variables are summarized with mean and standard deviation (SD), and categorical variables as frequency and percentage. Odds ratios and 95% confidence intervals were calculated from crude frequencies in contingency tables, using median-unbiased estimation and exact method. As this study aimed to provide only a descriptive analysis, no significance testing was performed. Initially, outcomes with missing values were imputed by taking the average of the outcome across all intervals excluding the baseline period. Comparisons between outcomes with and without imputation revealed no significant differences (data not shown). Therefore, we provide data without imputations for missing data.

Descriptive analyses were performed for subpopulations according to the total number of switches between anti-CGRP(-R) mAbs during the observation time - no switch, one switch, or two switches. Each treatment cycle with one type of antibody was considered when summarizing clinical outcome data. Clinical response to therapy during the first 6 months was summarized for each treatment cycle recorded in the observation period. The population is divided into patients who did not switch, switched once and switched twice. The outcomes of these groups are reported for each treatment cycle with one anti-CGRP(-R) mAb separately. For example, for the group of patients switching once, outcomes of the first and second treatment cycles are reported.

### Ethical review and regulatory considerations

The data acquisition and management protocol of the NTD platform was approved by the ethical committee of the Bavarian Medical Board (Bayerische Landesärztekammer; June 14, 2012) and re-approved by the ethical committee of the Medical Board North-Rhine (Ärztekammer Nordrhein, April 25, 2017). Compliance with European and German legislation (German Federal Data Protection Act, General Data Protection Regulation) is warranted including patient rights and informed consent requirements.

## Results

### Patient characteristics

Of the 5655 patients with the diagnosis of migraine in the NTD migraine registry, 670 patients (11.8%) received at least one anti-CGRP(-R) mAb for migraine prophylaxis. Of those, 655 patients met our inclusion criteria and were included in the study. The 15 excluded patients had either less than 4 MMDs at baseline or were less than 18 years old. The included patients were followed up for an average of 730 ± 470 days.

Baseline characteristics of the whole cohort, and by total number of switches during the observation period are displayed in Table 1. Of the 655 patients, 479 (73.1%) did not switch their antibody during the observation period, while 135 patients switched once (20.6%), 35 patients switched twice (5.4%), and 6 patients switched 3 or more times (0.9%). The average duration of a completed treatment cycle was 532 ± 438 days. For the no switch, one switch and two-switches subgroups, the average durations of the first completed treatment cycle were 679 ± 479, 338 ± 247, and 292 ± 243 days respectively. Data from the group of patients with 3 or more switches are not shown due to the low number of patients in this group.

**Table 1:** Baseline characteristics

		No switch (*n* = 479)	One switch (*n* = 135)	Two switches (*n* = 35)	Total^a)^ (*n* = 655)
Female, n (%)		427 (89.1%)	122 (90.4%)	31 (88.6%)	586 (89.5%)
Age, mean (SD)		47.2 (11.4)	46.6 (12.5)	47.6 (13.2)	47.1 (11.7)
Time since migraine diagnosis in years, mean (SD)		20.0 (13.6)	20.1 (13.8)	21.6 (13.0)	20.0 (13.7)
Chronic migraine, n (%)		65 (13.6%)	45 (33.3%)	14 (40.0%)	128 (19.5%)
Number of prior preventive failures, n (%)					
	1	22 (4.6%)	2 (1.5%)	2 (5.7%)	26 (4.0%)
	2	102 (21.3%)	48 (35.6%)	17 (48.6%)	170 (26.0%)
	3	75 (15.7%)	21 (15.6%)	4 (11.4%)	101 (15.4%)
	4	83 (17.3%)	21 (15.6%)	2 (5.7%)	107 (16.3%)
	4+	129 (26.9%)	28 (20.7%)	5 (14.3%)	163 (24.9%)
	Missing	68 (14.2%)	15 (11.1%)	5 (14.3%)	88 (13.4%)
Previous Botox therapy, n (%)		87 (18.2%)	40 (29.6%)	13 (37.1%)	145 (22.1%)
Education, n (%)					
	Technical college	39 (8.1%)	13 (9.6%)	4 (11.4%)	57 (8.7%)
	High school diploma	136 (28.4%)	43 (31.9%)	12 (34.3%)	193 (29.5%)
	Secondary general school	42 (8.8%)	13 (9.6%)	4 (11.4%)	59 (9.0%)
	No qualifications	1 (0.2%)	1 (0.7%)	0 (0.0%)	2 (0.3%)
	Polytechnic high school	14 (2.9%)	0 (0.0%)	0 (0.0%)	14 (2.1%)
	Intermediate secondary school	154 (32.2%)	48 (35.6%)	9 (25.7%)	214 (32.7%)
	Elementary school	4 (0.8%)	2 (1.5%)	3 (8.6%)	9 (1.4%)
	Missing	89 (18.6%)	15 (11.1%)	3 (8.6%)	107 (16.3%)
Employment, n (%)					
	Education	8 (1.7%)	4 (3.0%)	1 (2.9%)	14 (2.1%)
	Employed (full time)	156 (32.6%)	46 (34.1%)	12 (34.3%)	215 (32.8%)
	Employed (part time)	117 (24.4%)	34 (25.2%)	7 (20.0%)	161 (24.6%)
	Retired	40 (8.4%)	24 (17.8%)	10 (28.6%)	75 (11.5%)
	Homemaker	22 (4.6%)	6 (4.4%)	2 (5.7%)	30 (4.6%)
	Unemployed	14 (2.9%)	5 (3.7%)	1 (2.9%)	20 (3.1%)
	Missing	122 (25.5%)	16 (11.9%)	2 (5.7%)	140 (21.4%)
Comorbid psychiatric disorders, n (%)					
	Somatoform disorders	55 (11.5%)	33 (24.4%)	15 (42.9%)	103 (15.7%)
	Depression	116 (24.2%)	52 (38.5%)	7 (20.0%)	175 (26.7%)
	Anxiety disorder	22 (4.6%)	12 (8.9%)	3 (8.6%)	39 (6.0%)
Medication overuse, n (%)		106 (22.1%)	41 (30.4%)	9 (25.7%)	158 (24.1%)
MHD, mean (SD)		11.1 (5.5)	13.3 (6.3)	16.0 (7.4)	11.6 (6.1)
MMD, mean (SD)		9.9 (4.5)	12.3 (5.8)	14.5 (6.6)	10.7 (5.1)
MDAM, mean (SD)		8.1 (4.2)	9.2 (4.4)	8.7 (5.1)	8.7 (4.7)
HIT-6, mean (SD)		65.3 (4.1)	65.1 (4.9)	65.3 (3.6)	65.2 (4.3)
MIDAS, mean (SD)		62.7 (48.9)	67.6 (46.3)	57.3 (33.9)	62.4 (46.8)

Baseline characteristics of the whole cohort and subgroups with no switch, one switch, and two switches. SD: standard deviation, MHD: monthly headache days, MMD: monthly migraine days, MDAM: monthly days of acute medication. a) Total cohort includes the group with 3 or more switches (*n* = 6), which is not displayed separately in this table due to the small sample size

Patients who switched were more likely to have comorbid depression (OR: 1.74, 95% CI: 1.19–2.52), comorbid anxiety disorder (OR: 2.22, 95% CI: 1.13–4.30), medication overuse at baseline (OR: 1.60, 95% CI: 1.08–2.34), chronic migraine (OR 3.54, 95% CI: 2.36–5.32), and to have had previous prophylactic therapy with onabotulinumtoxinA (OR 2.21, 95% CI: 1.49–3.27), which in Germany is required for treatment with anti-CGRP mAbs in chronic migraine.

### Switching patterns

The highest number of patients (*n* = 427) initially received erenumab, which was the first available mAb in Germany. Of these, 134 (31.4%) went on to switch either to fremanezumab or galcanezumab. Twenty-two (14.2%) of the 150 patients who started with fremanezumab switched either to erenumab or galcanezumab, and 16 (20.5%) out of 78 patients who started with galcanezumab switched to either erenumab or fremanezumab. Most recorded switches (192 out of 221 switches, 86.9%) were either from an anti-CGRP-receptor to ligand mAb, or from an anti-CGRP-ligand to receptor mAb. Of the 172 first switches, only 9 (5.23%) were within the anti-CGRP ligand group. The number of patients receiving erenumab, fremanezumab or galcanezumab in their first up to sixth treatment attempt with an anti-CGRP(-R) mAb is displayed in Table 2.

**Table 2:** Number of patients receiving erenumab, fremanezumab or galcanezumab during their 1st -6th treatment cycles

	1st TC	2nd TC	3rd TC	4th TC	5th TC	6th TC	Total number of TCs
Erenumab	427	29	17	1	1	0	475
Fremanezumab	150	89	9	3	0	0	251
Galcanezumab	78	54	14	2	0	1	149

Number of patients receiving erenumab, fremanezumab or galcanezumab during their first to sixth treatment attempts (treatment cycles) with an anti-calcitonin gene-related peptide (-receptor) monoclonal antibody. TC: treatment cycle

**Fig. 1:**
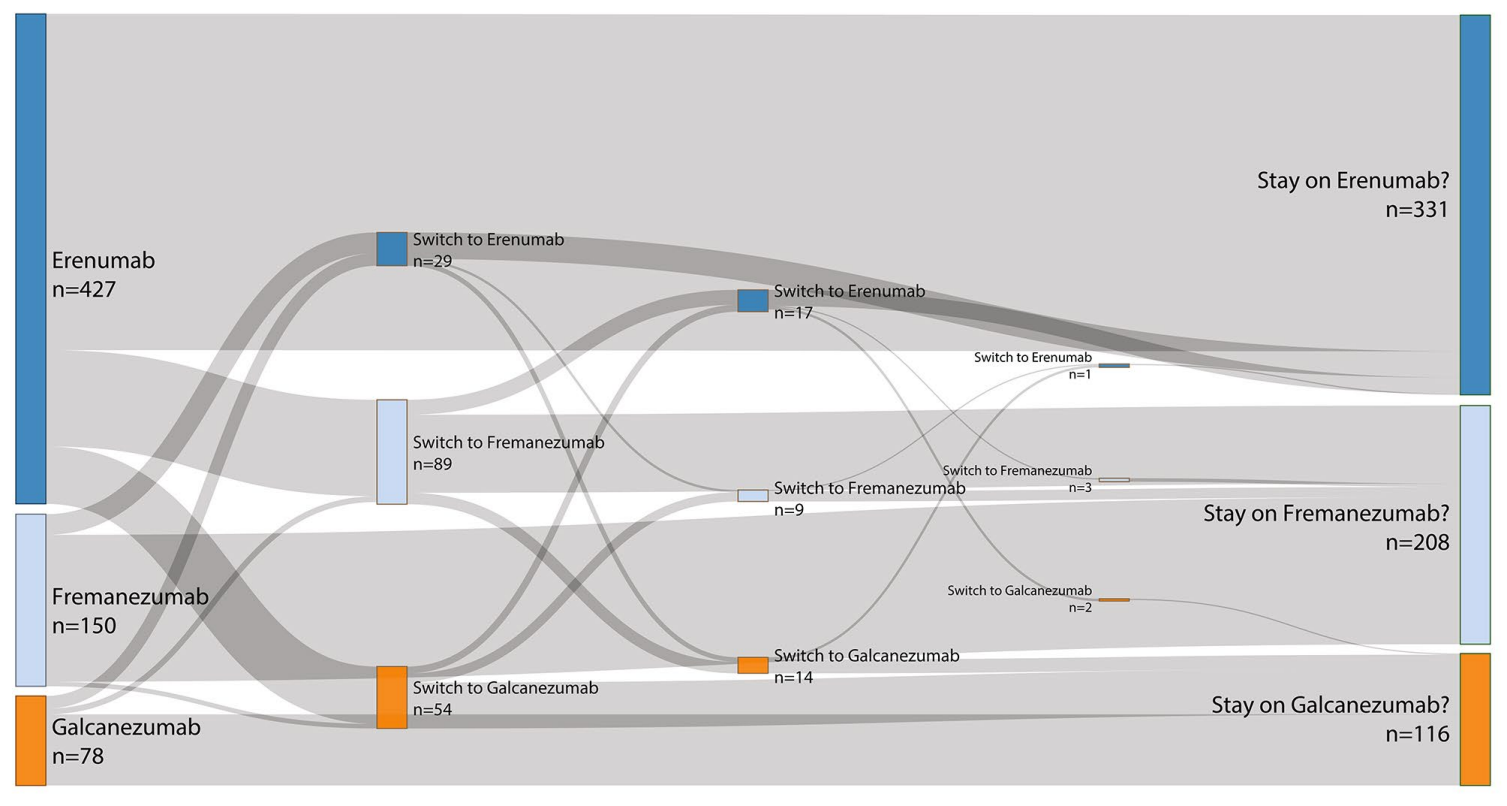
Sankey diagram of switching patterns among erenumab, fremanezumab and galcanezumab

Of the 6 patients who switched 3 times or more, 5 patients switched 3 times, and one patient switched 5 times. The maximum number of switches recorded was 5 times. A Sankey diagram of the switching pattern is shown in Fig. 1. The proportion of treatment cycles that were aborted before month 6 were 11.1% (53 out of 479 treatment cycles), 8.23% (22 out of 267 treatment cycles), and 13.3% (14 out of 105 treatment cycles) for the no switch, one switch, and two switches subgroups respectively.

### Clinical outcome

#### No switch subgroup

**Fig. 2:**
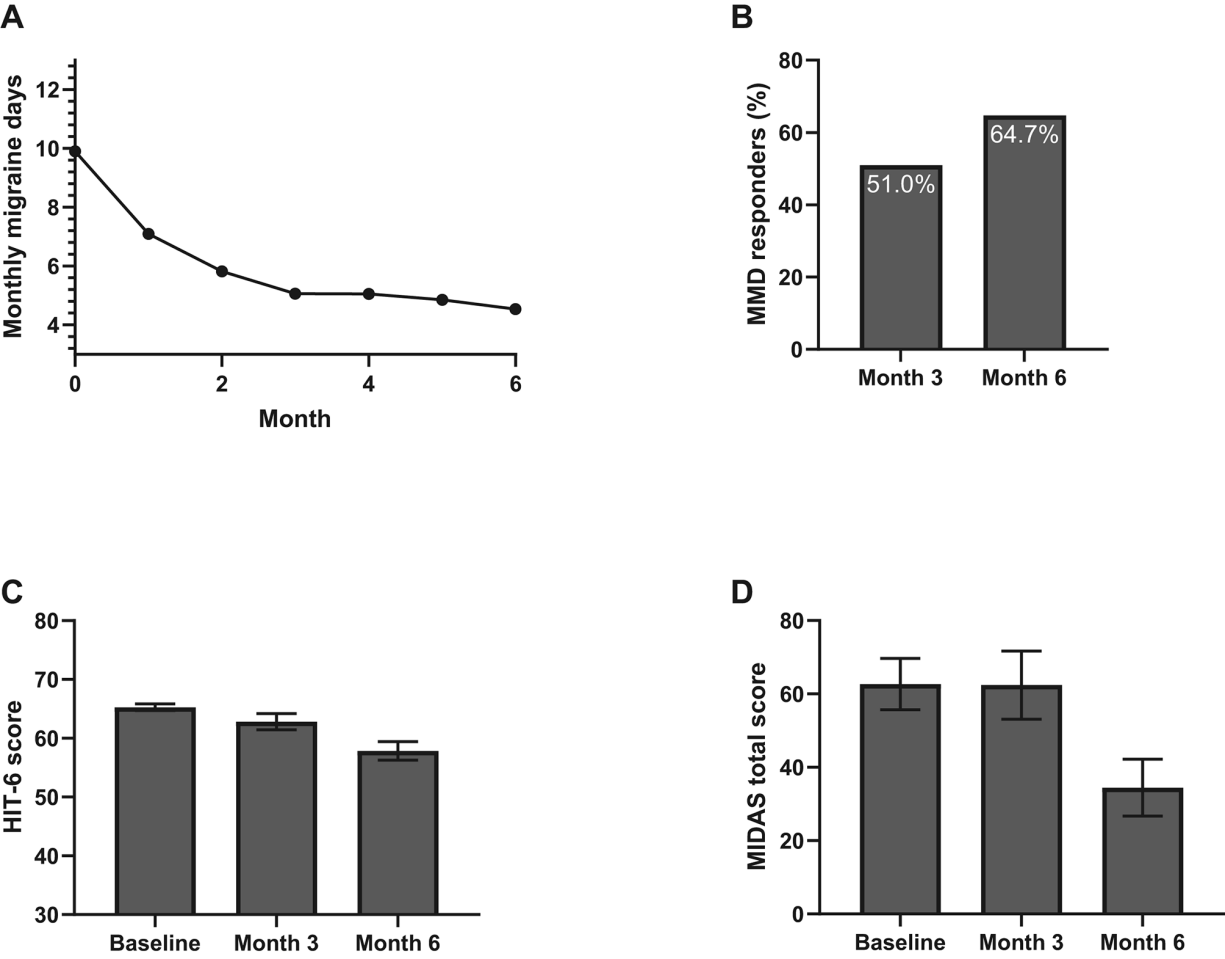
Results for the no switch subgroup. Mean monthly migraine days (MMD) during the first 6 months of treatment (**A**), percentage of patients achieving ≥ 50% reduction in MMD at 3 and 6 months (**B**), average HIT-6 (**C**) and MIDAS (**D**) scores at baseline, 3 and 6 months for the no switch subgroup. Error bars represent 95% confidence intervals

In patients who did not switch, a ≥ 50% reduction in MMD was achieved by 51.0% of patients at 3 months, and 64.7% of patients at 6 months (Fig. 2B). The mean MMD decreased from 9.90 (SD 4.50) days at baseline to 4.53 (SD 3.86) days by 6 months (change in mean MMD: -5.37, Fig. 2A).

MIDAS total scores improved from 62.7 (SD 48.9) at baseline to 34.5 (SD 42.4) at month 6 and HIT-6 scores improved from 65.3 (SD 4.09) to 57.9 (SD 8.60, Fig. 2C-D). Remaining results including MHD, ≥ 50% response rates for MHD, and MDAM during the first 6 months of therapy for all subgroups are displayed in Figures S1-S3.

#### One switch subgroup

**Fig. 3:**
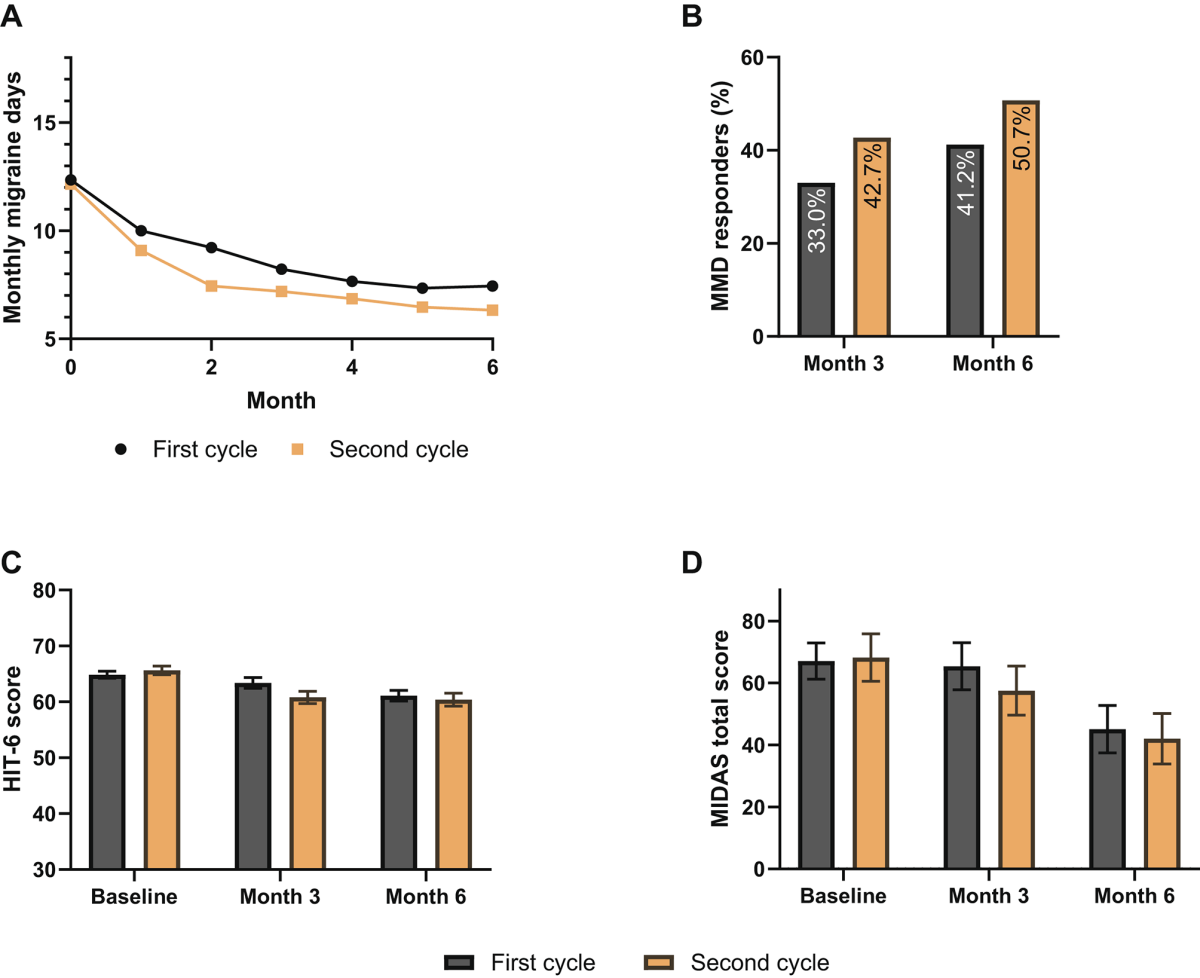
Results for the one switch subgroup. Mean monthly migraine days (MMD) during the first 6 months (**A**), percentage of patients achieving ≥ 50% reduction in MMD at 3 and 6 months (**B**), average HIT-6 (**C**) and MIDAS (**D**) scores at baseline, 3 and 6 months during the first and second treatment cycles of the one switch subgroup. Error bars represent standard errors of the mean

In the one switch subgroup, 33.0% and 41.2% of patients reached a ≥ 50% reduction in MMD at 3 and 6 months during the treatment cycle with the first anti-CGRP(-R) mAb. During the second treatment cycle after switching to a different anti-CGRP(-R) mAb, the ≥ 50% response rates for MMD were slightly higher with 42.7% at 3 months and 50.7% at 6 months (Fig. 3B). Mean MMD decreased from 12.3 (SD 5.85) at baseline to 7.44 (SD 5.33, change in mean MMD: -4.86) at 6 months in the first treatment cycle, and from 12.2 (SD 5.79) at baseline to 6.33 (SD 5.21, change in mean MMD: -5.87) at 6 months during the second treatment cycle (Fig. 3A).

From baseline to month 6 of therapy, MIDAS scores improved from 67.1 (SD 44.8) to 45.1 (SD 48.9) during the first treatment cycle, and from 68.2 (SD 48.9) to 42.0 (SD 47.3) during the second treatment cycle (Fig. 3D). HIT-6 scores changed from 64.9 (SD 5.10) to 61.1 (SD 6.04), and from 65.6 (SD 4.60) to 60.4 (SD 6.28) during the first 6 months of the first and second treatment cycles respectively (Fig. 3C).

#### Two switches subgroup

**Fig. 4:**
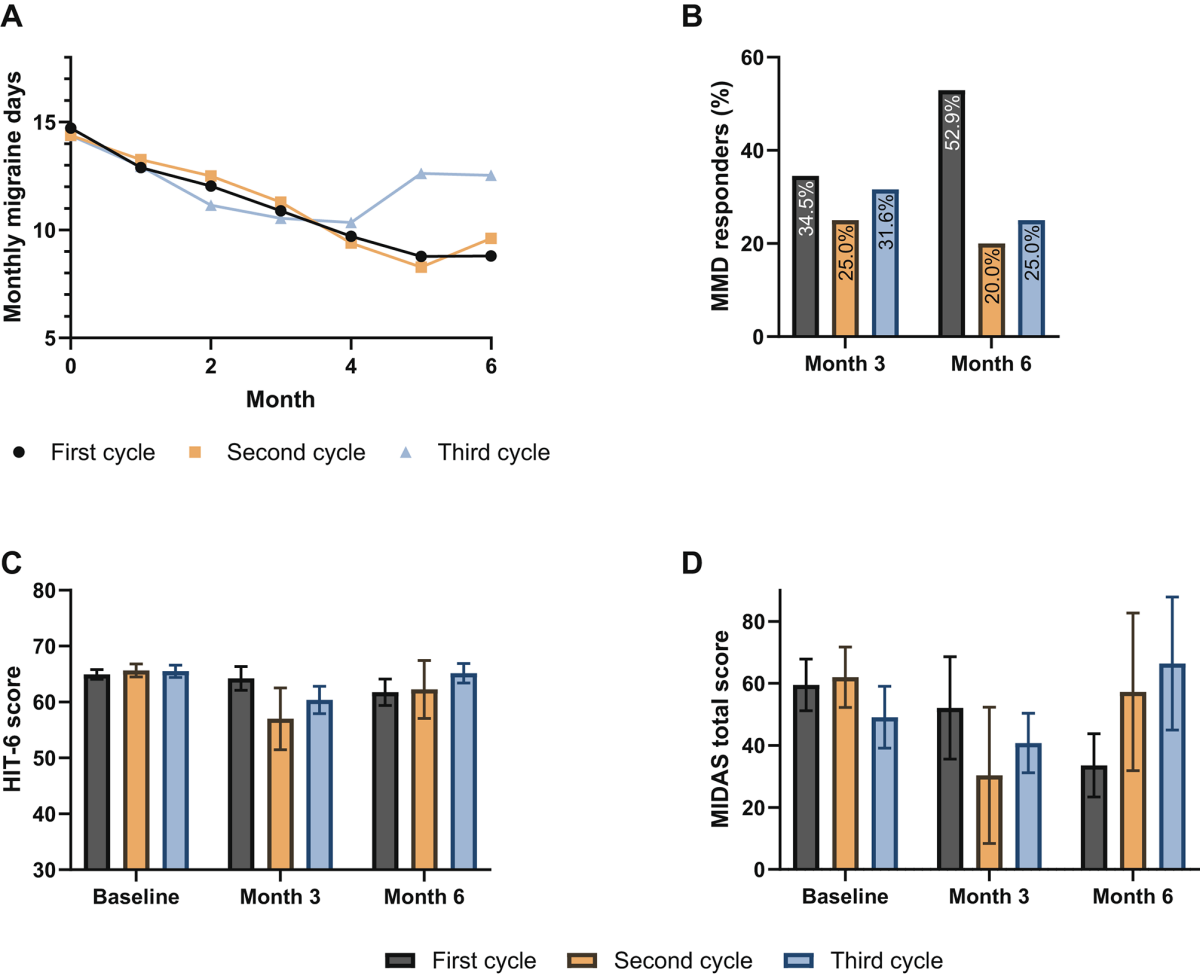
Results for the two switches subgroup. Mean monthly migraine days (MMD) during the first 6 months (**A**), percentage of patients achieving ≥ 50% reduction in MMD at 3 and 6 months (**B**), average HIT-6 (**C**) and MIDAS (**D**) scores at baseline, 3 and 6 months during the first, second, and third treatment cycles of the two switches subgroup. Error bars represent standard errors of the mean

In the group of patients who switched twice, the ≥ 50% response rates for MMD at month 3 were 34.5%, 25.0%, and 31.6% for the first, second and third treatment cycles respectively. At 6 months, the proportion of patients achieving a ≥ 50% reduction in MMD were 52.9%, 20.0% and 25.0% for the first, second and third treatment cycles respectively (Fig. 4B). Mean MMD decreased from 14.7 (SD 6.88) and 14.4 (SD 6.55) at baseline to 8.79 (SD 7.09, change in MMD − 5.91) and 9.61 (SD 3.71, change in mean MMD − 4.79) at 6 months during the first and second treatment cycles respectively. In the third treatment cycle, the decrease in mean MMD was lower, from 14.4 (SD 6.55) at baseline to 12.5 (SD 7.30, change in mean MMD − 1.84) at 6 months (Fig. 4A).

MIDAS and HIT-6 scores did not improve for the two switches subgroup during their treatment cycles (Fig. 4C-D). Mean MIDAS total scores at 6 months were even higher with increasing number of switches, with 33.6 (SD 30.6), 57.3 (SD 50.8), and 66.4 (SD 56.8) for the first, second and third treatment cycles respectively (Fig. 4D).

## Discussion

In this large cohort study, we observed that a significant proportion (26.9%) of migraine patients who received an anti-CGRP(-R) mAb, switched their antibody type at least once. Most switches involved changing antibody class (CGRP-R to CGRP mAb or vice versa), only a minority of switches were between CGRP ligand antibodies. Patients who switched at least once during treatment had higher baseline monthly headache and migraine days than those who did not have to switch. Switchers were more likely to have chronic migraine, comorbid depression and anxiety disorder and medication overuse, indicating an overall higher baseline disease burden, as well as the presence of risk factors for migraine chronification. The rates of ≥ 50% response in MHD and MMD were lower with increasing number of switches, which is in line with previous published reports from both real-world studies [11–13] and randomized controlled trials [14–17]. 

In real-world observational studies, the ≥ 50% response rates to anti-CGRP(-R) mAbs range from 50% to 70% depending on baseline patient characteristics and duration of therapy, leaving 30%–50% of patients not benefiting significantly from treatment. Accumulating recent reports of non-responders to anti-CGRP(-R) mAbs and switchers [6, 8, 9, 18, 19], highlight the fact that, while anti-CGRP(-R) mAbs are highly effective and well-tolerated medications, they are not the panacea patients sometimes hope for, and have relevant limitations. This could be due to alternative neuropeptides and signaling pathways involved in migraine pathogenesis playing a more crucial role in the non-responders [20, 21]. The presence of central sensitization and daily headaches as a consequence of migraine chronification is thought to be predictive of a non-response to anti-CGRP(-R) mAbs [22, 23]. Comorbidities such as depression or obesity that predispose to migraine chronification [24] were more common in non-responders to anti-CGRP(-R) mAbs [23, 25]. 

Our results show that, on average, patients who switch their antibody once, still benefit from the prophylactic treatment with anti-CGRP(-R) mAbs, with slightly better treatment results after the switch. In the one switch subgroup, the average ≥ 50% MMD response rates of the second treatment cycle after three and six months were 42.7% and 50.7%, respectively. This is comparable to ≥ 50% response rates seen in patients on their second anti-CGRP(-R) mAb in some other studies, e.g. 42.8% (59/138) in a real world study with fremanezumab (Finesse) at three months [19], and 42.3% (11/26) in an Austrian case series [9]. Other studies with limited sample sizes reported lower ≥ 50% response rates after switching: 13.6% (3/22) in Iannone et al. 2023 [8], 12% (3/25) and 5% (1/20) in Overeem et al. 2021 and 2023 [6, 7], 15.4% (6/39) in Lambru et al. 2023 [26], and 15% (10/66) in Talbot et al. 2024 [27]. 

In the group of patients with two switches, the ≥ 50% response rate and mean reduction in MHD and MMD at six months were distinctly lower, in particular during the third treatment cycle, while the baseline MHD and MMD were higher than in the other subgroups. While the mean MMD still fell by 5.93 and 4.77 days over the first 6 months of the first and second treatment cycles in this subgroup, that decrease was just 1.84 days during the third treatment cycle. Though the accuracy of these results may be affected by the small sample size of the two switches subgroup, it raises the question whether we should recommend a second antibody switch to our patients. As there are two classes of mAbs, either anti-CGRP mAb or anti-CGRP-R mAb, and the first switch mostly involves a change in antibody class, the second switch likely leads to a treatment with an antibody class that has already been tried. It would be interesting to see whether the treatment outcomes of the third treatment cycle would differ from the observations of our study, if the order of the antibody switch was reversed, and the second switch was the one involving a change to an antibody class that has not been previously tried. Further studies on the efficacy of specific antibody switches (for example switch from anti-CGRP-R mAb to anti-CGRP mAb or vice versa, or switches involving changes in route of administration) are needed to help guide treatment decisions.

Having to cycle through multiple antibodies puts a toll on the patients’ quality of life. In our study, we observed a worsening of headache impact and headache-related disability in patients who switched mAbs more than once. These results point to the need to address the overall headache burden when patients go through multiple treatment failures. It also highlights the potential harms of repeated therapy attempts that end in failure, with the patient cycling through phases of expectation followed by disappointment from treatment results.

Our study has several limitations that warrant consideration. The reasons for switching, whether due to side effects or lack of treatment response, were not reflected in the analyses, neither was our analysis adjusted for premature cessations. Another limitation is the disparate size of the subgroups of interest, with the majority of our cohort not switching their antibody. Moreover, the use of concomitant preventive medication was not assessed, potentially influencing the observed outcomes. However, it is worth noting that in Germany monotherapy is typically recommended, and the prevalence of patients receiving concomitant preventive treatments is expected to be low. Finally, in the data provided, especially in the case of patient reported outcome measures, there was a significant portion of missing data, contributing to the uncertainty of the estimates, and possibly introducing a systematic bias in those who provided responses.

Despite these limitations, the database provides detailed high-quality descriptive data on an emerging patient population that is challenging to treat and bound to grow in the future. While initial switches appear to offer some benefit, multiple switches may lead to diminishing treatment effectiveness and increased burden on patients, emphasizing the importance of carefully considering treatment strategies in this population.

### Electronic supplementary material

Below is the link to the electronic supplementary material.

Supplementary Material 1

## Data Availability

The data used in this study are owned by the NeuroTransData Registry and sharing of the data is subject to their policies. Any reasonable requests for data access can be directed to the NeuroTransData Registry (www.neurotransdata.com).
