# Supplementary material for: Therapeutic patterns and migraine disease burden in switchers of CGRP-targeted monoclonal antibodies – insights from the German NeuroTransData registry
Source: J Headache Pain. 2024 Jun 3;25(1):90. doi: 10.1186/s10194-024-01790-7 (PMC11145812; doi:10.1186/s10194-024-01790-7)
Supplement: Supplementary file 1 — Supplementary Material 1 [file 10194_2024_1790_MOESM1_ESM.docx]

**Supplemental Material**


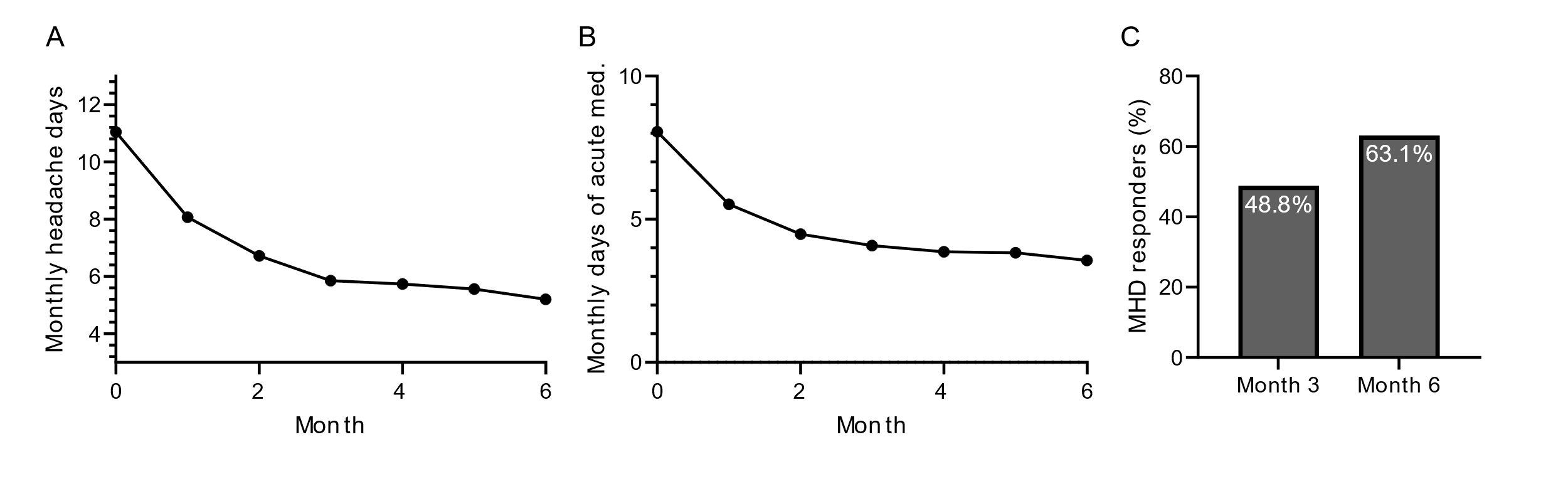


**Figure S1.** **Supplemental results of the no switch subgroup.** Mean monthly headache days (MHD, A) and monthly days acute medication (B) during the first 6 months of treatment and percentage of patients achieving ≥50% reduction in MHD at 3 and 6 months (C).


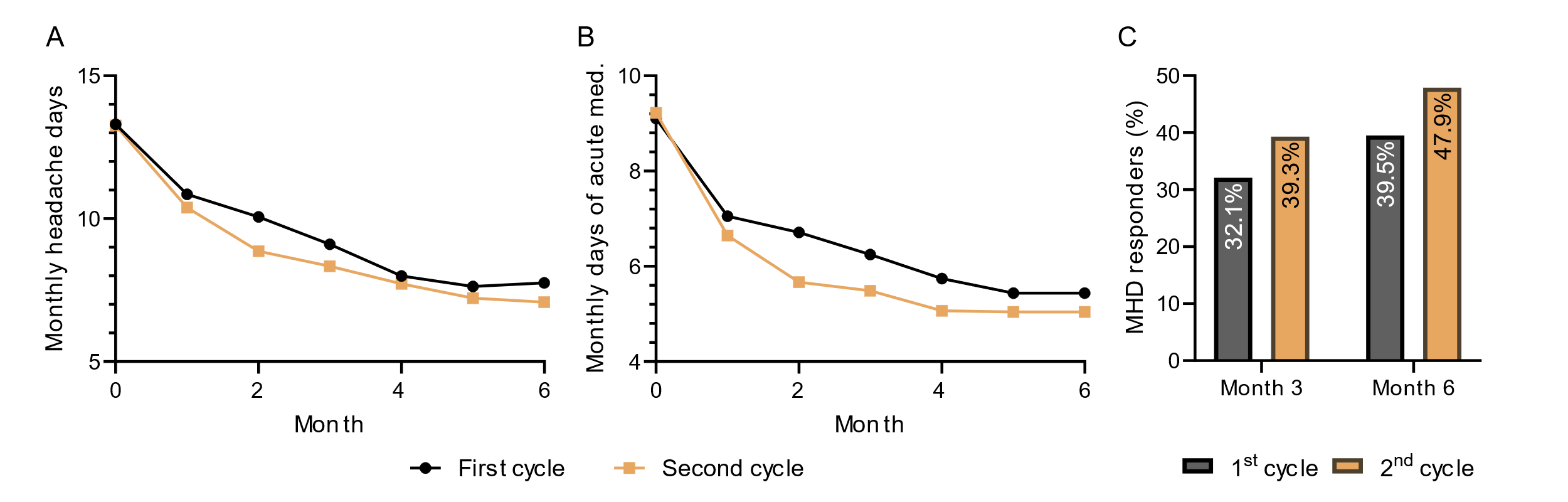


**Figure S2. Supplemental results of the one switch subgroup.** Mean monthly headache days (MHD, A) and monthly days acute medication (B) during the first 6 months of the first and second treatment cycle and percentage of patients achieving ≥50% reduction in MHD at 3 and 6 months of the first and second treatment cycle (C).


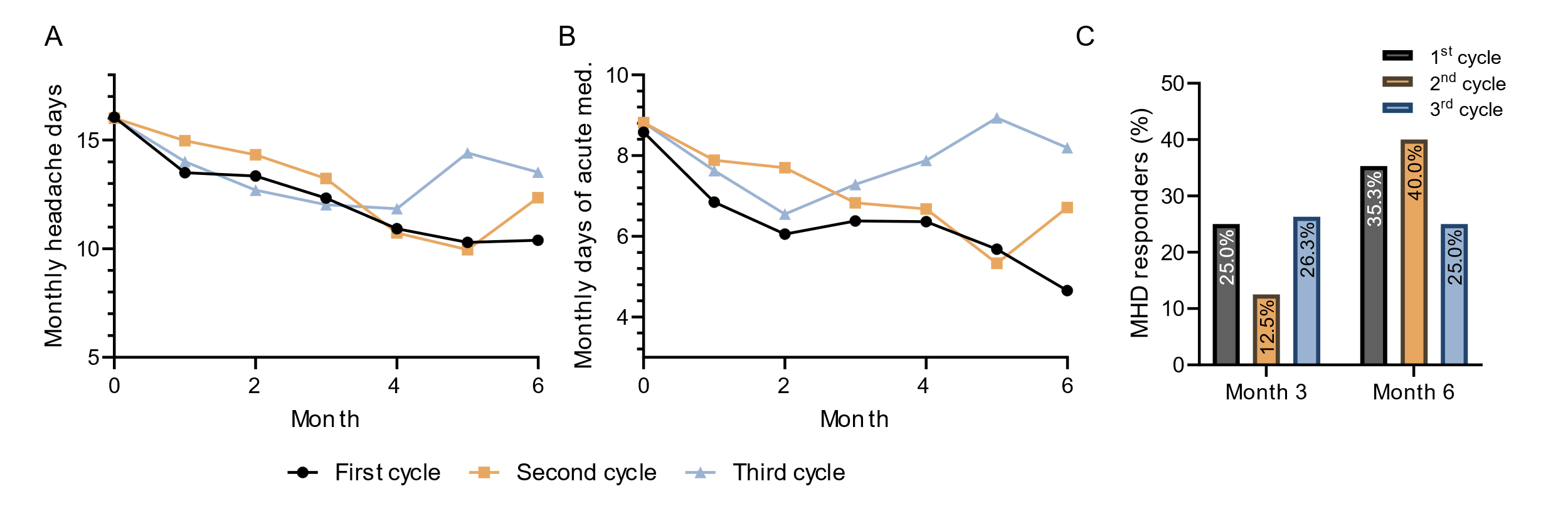


**Figure S3. Supplemental results of the two switches subgroup.** Mean monthly headache days (MHD, A) and monthly days acute medication (B) during the first 6 months of the first, second and third treatment cycle and percentage of patients achieving ≥50% reduction in MHD at 3 and 6 months of the first, second and third treatment cycle (C).
